# Current knowledge and practice of Australian and New Zealand health‐care professionals in sarcopenia diagnosis and treatment: Time to move forward!

**DOI:** 10.1111/ajag.12730

**Published:** 2019-10-15

**Authors:** Suey S. Y. Yeung, Esmee M. Reijnierse, Marijke C. Trappenburg, Carel G. M. Meskers, Andrea B. Maier

**Affiliations:** ^1^ Department of Medicine and Aged Care, @AgeMelbourne The Royal Melbourne Hospital The University of Melbourne Melbourne Victoria Australia; ^2^ Department of Human Movement Sciences, @AgeAmsterdam Faculty of Behavioural and Movement Sciences Amsterdam Movement Sciences Vrije Universiteit Amsterdam The Netherlands; ^3^ Department of Internal Medicine Section of Gerontology and Geriatrics Amsterdam UMC Vrije Universiteit Amsterdam The Netherlands; ^4^ Department of Internal Medicine Amstelland Hospital Amstelveen The Netherlands; ^5^ Department of Rehabilitation Medicine Amsterdam UMC Vrije Universiteit Amsterdam Movement Sciences Amsterdam The Netherlands

**Keywords:** diagnosis, health personnel, knowledge, sarcopenia, therapy

## Abstract

**Objectives:**

To describe the current knowledge and practice of sarcopenia diagnosis and treatment among health‐care professionals before, directly after and 6 months after a professional development event on sarcopenia.

**Methods:**

This longitudinal study included Australian and New Zealand health‐care professionals who completed questionnaires on knowledge, practice and barriers regarding sarcopenia before, directly after and 6 months after attending a professional development event on sarcopenia.

**Results:**

A total of 250 professionals participated; 84 completed the 6‐month questionnaires. Before, directly after and at 6 months, respectively, 14.7%, 93.4% and 59.5% identified sarcopenia as a disease; 2.0%, 79.6% and 38.1% correctly answered the sex‐specific cut‐offs for low handgrip strength. Respectively, 12.0% and 14.3% reported to make sarcopenia diagnoses as part of their practice before and at 6 months.

**Conclusions:**

Knowledge about sarcopenia is limited among health‐care professionals who attended a professional development event. Retention of knowledge remains a challenge to be addressed.

**Policy Impact**

Intention to diagnose sarcopenia contrasts the practice in diagnosing sarcopenia. There is an opportunity to facilitate changes in practice regarding sarcopenia diagnosis and treatment among health‐care professionals. This may include the provision of diagnostic tools, development of treatment protocols and increase in awareness among health‐care professionals.

**Practice Impact**

Health‐care professionals’ knowledge improved after a single lecture on sarcopenia, but retention of knowledge remains a challenge to be addressed. At the individual level, health‐care professionals should actively engage in continuous professional development to acquire up‐to‐date knowledge.

## 1 INTRODUCTION

The presence of low muscle mass and function^1^ is termed sarcopenia, which is present in 10% of community‐dwelling older adults.^2^ Adverse health outcomes of sarcopenia include functional decline, falls, fractures, hospitalisation and mortality.^3^, ^4^ Sarcopenia is estimated to increase hospitalisation costs by 34% for older adults^5^ and to contribute 1.5% of total health‐care costs in the United States.^6^ With the increase in life expectancy, sarcopenia becomes a global public health problem.^7^ Like many other diseases, sarcopenia is asymptomatic in its initial stage.^8^ Therefore, early diagnosis and subsequent intervention are essential. For this, awareness among health‐care professionals is a prerequisite.

Most Dutch health‐care professionals reported to know what sarcopenia is.^9^ Among 683 members from the Japanese Association of Rehabilitation Nutrition, including mainly dietitians and physiotherapists working in acute general wards and convalescent rehabilitation wards, less than half (42%) measured all items necessary for the diagnosis of sarcopenia, including muscle mass, muscle strength and physical performance.^10^ Because of the difference in health‐care systems,^11^ findings from the aforementioned studies cannot directly be translated to other countries. In addition, no study has reported the retention of actual knowledge after a professional development event on sarcopenia. There is also a need to further address knowledge gaps in relation to possible barriers to diagnosing and treating sarcopenia in daily clinical practice.

The primary aim of this study was to describe the current knowledge and practice regarding sarcopenia. Secondary aims were to assess the changes in knowledge and practice 6 months after attending a professional development event on sarcopenia and to identify barriers in diagnosing and treating sarcopenia in a cohort of Australian and New Zealand health‐care professionals.

## 2 METHODS

### 2.1 Study design

This longitudinal study included allied health professionals and physicians who attended a professional development event (“Sarcopenia Roadshow”). It was held at six locations in Australia and New Zealand (ie Sydney, Melbourne, Auckland, Tauranga, Palmerston and Christchurch) between September 2017 and October 2017. Sarcopenia Roadshow was advertised via local hospitals and community health services, and attendance was free of charge. Immediately before and directly after the events, attending health‐care professionals were invited to complete the questionnaires. Six months after attendance, a follow‐up questionnaire was sent by email to health‐care professionals who agreed to be contacted for follow‐up. Reminders were sent 2 weeks after the initial request. Ethical guidelines were followed in accordance with the Declaration of Helsinki. No medical ethical approval was required.

### 2.2 Sarcopenia Roadshow

The Sarcopenia Roadshow was a 2‐hour lecture series delivered by a geriatrician and a senior dietitian to increase the awareness of sarcopenia among health‐care professionals. The lecture encompassed the presentation of the following topics: ageing trajectory of muscle mass, the European Working Group on Sarcopenia in Older People (EWGSOP) 2010 definition^12^ and diagnostic tools commonly used to measure muscle mass, muscle strength and gait speed in clinical practice. Treatment of sarcopenia focused on non‐pharmacological interventions including resistance exercise and adequate protein intake. The rationale for a purely didactic delivery is based on the awareness‐to‐adherence model.^13^ According to this model, to comply with a new practice, health‐care professionals have to first become aware of the practice, then move to a process of agreement with it and then decide to adopt it in the care they provide, followed by adhering to the practice.^14^ Since sarcopenia has been recently recognised as a disease,^15^, ^16^, ^17^ raising awareness by means of traditional modalities, such as lectures, may be an effective initial step in predisposing health‐care professionals to change their practices.

### 2.3 Questionnaires

A structured questionnaire was developed and modified from a previous study.^9^ Face validity was tested among five allied health professionals and four physicians to ensure the questionnaire was easily understood. The questions included demographics (age, sex, profession, years of practice and working affiliation), knowledge about sarcopenia diagnostic strategy and treatment, and practices in diagnosing and treating sarcopenia. The first part of the questionnaire aimed to describe the current knowledge and practice of sarcopenia before attendance. The second part aimed to examine the intention to implement diagnostic strategies and treat sarcopenia in clinical practice directly after attendance. The third part aimed to assess whether the knowledge was retained and whether there was any change in practice regarding diagnosing and treating sarcopenia 6 months after attendance. The questionnaires are presented in Appendix S1.

### 2.4 Statistical analyses

Data were analysed using Statistical Package for the Social Sciences, version 24.0 (SPSS Inc). Continuous variables were checked for normality and presented as mean (standard deviation [SD]) if normally distributed and as median (interquartile range [IQR]) if skewed. Categorical variables were presented as number (n) and percentage (%). *t* tests and chi‐square tests were used to compare the characteristics of health‐care professionals who did and did not complete the follow‐up questionnaires and between health‐care professionals dependent on their knowledge of sarcopenia. Visualisation of results was performed using GraphPad Prism 5.01.

## 3 RESULTS

### 3.1 Characteristics of attending health‐care professionals

A total of 287 health‐care professionals attended the lectures, and 250 (87%) responded to the questionnaires. Six months after attendance, questionnaires were sent out to 194 health‐care professionals who agreed to be contacted for follow‐up, of whom 16 could not be contacted. Of the 178 health‐care professionals whom we successfully contacted, 84 (47.2%) completed the follow‐up questionnaires. Table 1 shows the characteristics of health‐care professionals. The median [IQR] age was 40 [28‐55] years, and 83.8% were female, 59.8% were dietitians, 22.0% physicians, 14.6% nurses and 3.7% other disciplines. The median [IQR] years of practice was 10 [3‐30] years. The characteristics of those who completed the follow‐up questionnaires were not significantly different from those who did not, except for having received sarcopenia‐related education in the last 6 months.

**Table 1:** Characteristics of attending health‐care professionals (n = 250), stratified by those who did and did not complete the follow‐up questionnaires

	N	Total (n = 250)	FU	
			Completed (n = 84)	Did not complete (n = 166)
Age, y, median [IQR]	230	40 [28‐55]	40 [28‐54]	41 [27‐56]
Female	241	202 (83.8)	71 (86.6)	131 (82.4)
Profession	246			
Dietitian		147 (59.8)	54 (65.1)	93 (57.1)
Physician		54 (22.0)	16 (19.3)	38 (23.3)
Nurses		36 (14.6)	11 (13.3)	25 (15.3)
Others		9 (3.7)	2 (2.4)	7 (4.3)
Years of practice, median [IQR]	245	10 [3‐30]	10 [3‐26]	11 [3‐30]
Setting	242			
Community service		36 (14.9)	15 (18.3)	21 (13.1)
General practice		65 (26.9)	17 (20.7)	48 (30.0)
Outpatient clinic		23 (9.5)	8 (9.8)	15 (9.4)
Nursing home		20 (8.3)	6 (7.3)	14 (8.8)
Hospital		132 (54.5)	46 (56.1)	86 (53.8)
Other settings		29 (12.0)	7 (8.5)	22 (13.8)
Work with patients aged ≥65 y, yes	241	235 (97.5)	77 (96.3)	158 (98.1)
Received sarcopenia‐related education in the last 6 mo	242	41 (16.9)	20 (24.7)	21 (13.0)

Note

Variables are presented as n (%) unless indicated otherwise.

Abbreviations: FU, follow‐up; IQR, interquartile range; y, years; mo, months.

### 3.2 Knowledge about sarcopenia

Table 2 shows the current knowledge about sarcopenia among health‐care professionals. Before, directly after and 6 months after attendance, respectively, 14.7%, 93.4% and 59.5% of the professionals correctly stated that sarcopenia is a disease. Respectively, 73.6%, 84.5% and 83.3% correctly disagreed with the statement “Sarcopenia cannot be prevented,” and 73.3%, 92.1% and 88.1% correctly disagreed with the statement “Overweight or obese individuals have a lower risk of sarcopenia compared to individuals with normal weight.”

**Table 2:** Knowledge about sarcopenia, its diagnostic strategy and treatment among health‐care professionals before, directly after and 6 months after attendance

	Before		Directly after		6 mo after
	Total (n = 250)	Completed FU^a^ (n = 84)	Total (n = 250)	Completed FU^a^ (n = 84)	Completed FU^a^ (n = 84)
Knowledge about the concept					
Sarcopenia is recognised as a…					
Disease	34 (14.7)	12 (15.6)	228 (93.4)	80 (95.2)	50 (59.5)
Syndrome	33 (14.2)	12 (15.6)	1 (0.4)	0	13 (15.5)
Condition	138 (59.5)	45 (58.4)	15 (6.1)	4 (4.8)	21 (25.0)
Don't know	27 (11.6)	8 (10.4)	0	0	0
Sarcopenia cannot be prevented.					
Agree	41 (17.2)	12 (14.8)	36 (15.1)	13 (15.7)	12 (14.3)
Disagree	176 (73.6)	60 (71.4)	202 (84.5)	70 (84.3)	70 (83.3)
Don't know	22 (9.2)	9 (11.1)	1 (0.4)	0	2 (2.4)
Overweight or obese individuals have a lower risk of sarcopenia compared to individuals with normal weight.					
Agree	21 (8.9)	5 (6.3)	5 (2.1)	1 (1.2)	7 (8.3)
Disagree	173 (73.3)	58 (73.4)	222 (92.1)	81 (96.4)	74 (88.1)
Don't know	42 (17.8)	16 (20.3)	14 (5.8)	2 (2.4)	3 (3.6)
Diagnostic strategy					
Which criteria should be used to diagnose sarcopenia?					
Clinical impression	112 (47.3)	32 (40.0)	22 (8.9)	6 (7.1)	24 (28.6)
Muscle mass	213 (89.9)	70 (87.5)	231 (93.9)	80 (95.2)	66 (78.6)
Muscle strength	193 (81.4)	67 (83.8)	241 (96.4)	82 (97.6)	77 (91.7)
Physical performance	142 (59.9)	60 (75.0)	218 (88.6)	75 (89.3)	75 (89.3)
Nutritional status	147 (62.0)	44 (55.0)	77 (31.3)	20 (23.8)	28 (33.3)
Body mass index	77 (32.5)	23 (28.7)	13 (5.3)	4 (4.8)	14 (16.7)
Frailty index	149 (62.9)	46 (57.5)	34 (13.8)	13 (15.5)	30 (35.7)
Others	8 (3.4)	3 (3.8)	15 (6.1)	5 (6.0)	1 (1.2)
At what age do muscle mass and muscle strength start to decline? y, median [IQR]	50 [35‐60]	50 [30‐60]	25 [25‐30]	25 [25‐30]	30 [26‐35]
What is the cut‐off for low handgrip strength?					
For men, kg, median [IQR]	20 [10‐30]	20 [8‐30]	30 [30‐30]	30 [30‐30]	30 [27‐30]
Correct answer	11 (4.4)	3 (3.6)	206 (82.4)	71 (88.8)	33 (39.2)
For women, kg, median [IQR]	14 [6‐25]	12 [6‐23]	20 [20‐20]	20 [20‐20]	20 [20‐20]
Correct answer	6 (2.4)	1 (1.2)	202 (80.8)	69 (86.3)	33 (39.2)
Both answers correct	5 (2.0)	1 (1.2)	199 (79.6)	69 (82.1)	32 (38.1)
Treatment of sarcopenia					
Sarcopenia should be treated with…					
Physical exercise	225 (94.9)	76 (93.8)	244 (99.6)	84 (100)	84 (100)
Aerobic exercise	69 (31.5)	23 (31.1)	88 (35.2)	27 (32.1)	19 (22.6)
Resistance exercise	194 (88.6)	68 (91.9)	240 (99.6)	84 (100)	83 (98.8)
Balance exercise	106 (48.4)	36 (48.6)	116 (48.1)	38 (45.2)	39 (46.4)
Nutritional intervention	228 (95.8)	77 (95.1)	239 (97.6)	82 (97.6)	82 (97.6)
Protein	209 (95.4)	72 (97.3)	235 (97.5)	82 (97.6)	82 (97.6)
Vitamin D	123 (56.2)	49 (66.2)	219 (90.9)	75 (89.3)	62 (73.8)
Calcium	93 (42.5)	34 (45.9)	199 (82.6)	70 (83.3)	52 (61.9)
Pharmacological intervention	51 (21.4)	18 (22.2)	28 (11.4)	11 (13.1)	1 (1.2)
Don't know	8 (3.4)	2 (2.5)	0	0	0

Note

Variables are presented as n (%) unless indicated otherwise.

Abbreviations: FU, follow‐up; IQR, interquartile range; y, years; mo, months.

a Among health‐care professionals who completed the follow‐up questionnaires 6 mo after attendance.

Muscle mass, muscle strength and physical performance were correctly identified as diagnostic criteria of sarcopenia by 89.9%, 81.4% and 59.9% of the health‐care professionals, respectively. Health‐care professionals overestimated the age at which muscle mass and muscle strength start to decline, from a median age of 50 years [35‐60] before, to an estimation closer to the correct answer of 30 years directly after (25 years [25‐30]) and 6 months after attendance (30 years [26‐35]). Sex‐specific cut‐off points for low handgrip strength were correctly answered by 2.0%, 79.6% and 38.1% of the health‐care professionals before, directly after and 6 months after attendance, respectively.

Resistance exercise and adequate protein intake were correctly identified as sarcopenia treatment by about 90% of health‐care professionals. There was a substantial decrease in the percentage of health‐care professionals who thought that sarcopenia should be treated with pharmacological intervention, from 21.4% before to 11.4% directly after and 1.2% 6 months after attendance.

Professionals who work in community services and received previous sarcopenia‐related education had significantly better knowledge about sarcopenia before attendance of the Sarcopenia Roadshow (Appendix S2).

The knowledge about sarcopenia among health‐care professionals who completed the follow‐up questionnaires was similar to those who had no follow‐up.

### 3.3 Sarcopenia diagnosis in clinical practice

**Figure 1:**
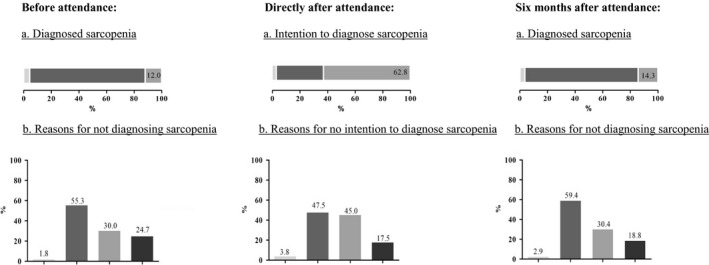
Sarcopenia diagnosis in clinical practice among health‐care professionals before (n = 250), directly after (n = 250) and 6 months after attendance (n = 84). A, (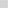) didn’t respond; (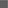) no; (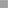) yes. B, (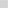) do not work with older adults; (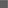) do not have the tools; (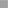) not responsible for diagnosing; (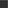) other reasons

Figure 1 shows the sarcopenia diagnosis in clinical practice before, directly after and 6 months after attendance. Appendix S3 presents the number of health‐care professionals responding to different questions regarding sarcopenia diagnosis in clinical practice. Twelve per cent of the health‐care professionals reported to make sarcopenia diagnoses as part of their practice before attendance. Although 62.8% intended to diagnose sarcopenia, only 14.3% reported to make sarcopenia diagnoses as part of their practice at 6 months of follow‐up. Lack of diagnostic tools was reported to be the main reason for not diagnosing sarcopenia (55.3% and 59.4% before and 6 months after attendance, respectively). Another frequently reported reason both before and 6 months after attendance (30.0% and 30.4%, respectively) was that professionals thought it was not their role to diagnose sarcopenia. Nurses were the main professional group (52.0%) who thought that it was not their role to diagnose sarcopenia, followed by dietitians (28.2%) and physicians (9.3%). Appendix S4 shows the diagnostic criteria and definition used by health‐care professionals who diagnosed sarcopenia. Muscle mass, muscle strength and physical performance were the least frequently used diagnostic criteria before attendance. Although over half of the health‐care professionals intended to use these diagnostic criteria directly after attendance, the number of health‐care professionals who used these diagnostic criteria remained low at 6 months of follow‐up. Of the health‐care professionals reporting the use of muscle mass as a diagnostic criterion at follow‐up, more than half used inappropriate methods such as calf circumference and skinfold thickness. Less than half of the health‐care professionals applied the diagnostic criteria to all older adults. Among health‐care professionals who diagnosed sarcopenia, the use of the main operational definitions of sarcopenia (ie EWGSOP 2010,^12^ International Working Group on Sarcopenia [IWGS]^18^ and Janssen 2004^19^) was low, and instead, inappropriate definitions (ie European Society for Parenteral and Enteral Nutrition malnutrition definition, frailty by Fried and frailty by Rockwood) were applied before and 6 months after attendance.

### 3.4 Sarcopenia treatment in clinical practice

Table 3 shows the sarcopenia treatment in clinical practice before and 6 months after attendance. For those who were responsible to provide sarcopenia treatment, there was an increase in providing resistance exercise from 12.4% before attendance to 28.6% at 6 months of follow‐up, while adequate protein intake was provided to patients diagnosed with sarcopenia by most of the health‐care professionals. When diagnosed, 51.7% stated to consult other disciplines before attendance, with a slight increase to 62.9% at follow‐up. Overall, the reported rate of consultation with physicians including general practitioners and specialists increased from 58.7% before attendance to 70.6% at 6 months of follow‐up. Responses of health‐care professionals who completed the follow‐up questionnaires were similar to all the health‐care professionals.

**Table 3:** Sarcopenia treatment in clinical practice among health‐care professionals before and 6 mo after attendance

	Before		6 mo after
	Total (n = 250)	Completed FU^a^ (n = 84)	Completed FU^a^ (n = 84)
Responsible for providing sarcopenia treatment, yes	141/222 (63.5)	48/75 (64.0)	42/82 (50.0)
Physical exercise	25 (18.5)	6 (12.8)	13 (31.0)
Aerobic	9 (7.0)	1 (2.2)	5 (11.9)
Resistance	16 (12.4)	5 (10.9)	12 (28.6)
Balance	11 (8.5)	3 (6.5)	9 (21.4)
Nutritional intervention	129 (96.3)	45 (95.7)	42 (100)
Protein	89 (91.8)	35 (94.6)	42 (100)
Vitamin D	53 (54.6)	21 (56.8)	27 (64.3)
Calcium	47 (48.5)	21 (56.8)	29 (69.0)
Consult with other disciplines when there is a patient diagnosed with sarcopenia, yes	109/211 (51.7)	34/68 (50.0)	17/27 (62.9)
Dietitian	59 (54.1)	16 (48.5)	9 (52.9)
Exercise physiologist	40 (36.7)	16 (48.5)	3 (17.6)
Physician	64 (58.7)	21 (63.6)	12 (70.6)
Nurse	22 (20.2)	7 (21.2)	2 (11.8)
Occupational therapist	29 (26.6)	8 (24.2)	1 (5.9)
Physiotherapist	79 (72.5)	22 (66.7)	11 (64.7)
Podiatrist	5 (4.6)	2 (6.1)	0
Others	2 (1.8)	0	1 (5.9)

Note

Variables are presented as n (%).

Abbreviation: FU, follow‐up; mo, months.

a Among health‐care professionals who completed the follow‐up questionnaires 6 mo after attendance.

### 3.5 Barriers in sarcopenia diagnosis and treatment

Table 4 shows the barriers reported by health‐care professionals. Five out of the 12 health‐care professionals, who diagnosed sarcopenia at 6 months of follow‐up, perceived to experience barriers during diagnosis. Lack of diagnostic tools (n = 3) and time constraints (n = 3) were reported to be the main barriers.

**Table 4:** Barriers reported by health‐care professionals 6 months after attendance

	N (%)
Barriers during diagnosis of sarcopenia (n = 12), yes	5 (41.7)
Acquisition of a device to measure muscle mass	3 (60.0)
Not trained to measure muscle mass	2 (40.0)
Acquisition of handgrip strength device	2 (40.0)
Do not have the skill in measuring handgrip strength	1 (20.0)
Time constraints to perform the diagnostic tests	3 (60.0)
No specific funding source for sarcopenia	1 (20.0)
Barriers during implementation of treatment plan (n = 42), yes	25 (59.5)
Restructuring of routine care	5 (20.0)
Lack of awareness among health‐care professionals	14 (56.0)
Lack of collaboration among health‐care professionals	4 (16.0)
No treatment protocol	19 (76.0)
Not a priority	9 (36.0)
Patient refused to be treated	3 (12.0)
Patient not aware of the treatment importance	7 (28.0)
Others	7 (28.0)
Barriers in treating patients (n = 42), yes	17 (40.5)
No access to other experienced health‐care professionals	4 (23.5)
Lack of awareness among health‐care professionals	8 (47.1)
Lack of motivation among health‐care professionals	6 (35.3)
Patients not motivated to be treated	4 (23.5)
Patients not compliant to treatment plan	5 (29.4)
Financial implications of treatment for patient	4 (23.5)
Not enough manpower to treat	4 (23.5)
Others	4 (23.5)

Of the 42 professionals who treated sarcopenia at 6 months of follow‐up, n = 25 perceived to experience barriers during implementing the treatment plan. Lack of treatment protocol (n = 19) and awareness among health‐care professionals (n = 14) were the most frequently perceived barriers. Seventeen health‐care professionals perceived to experience barriers during the actual treatment, of which lack of awareness (n = 8) and lack of motivation (n = 6) were the most common ones.

## 4 DISCUSSION

Knowledge about sarcopenia and its diagnostic strategy is limited among Australian and New Zealand health‐care professionals attending a professional development event. Knowledge was not retained 6 months after lecture attendance. Although over half of the professionals intended to diagnose sarcopenia directly after attendance, the practice of diagnosing sarcopenia remained low at 6 months of follow‐up. Lack of diagnostic tools and time constraints were reported as the main barriers.

### 4.1 Knowledge about sarcopenia

Previous studies from Australia^20^ and European countries^21^ found that respectively, 46% and 74% of dietitians correctly recorded sarcopenia as a diagnosis in a case study. Given that sarcopenia has been recognised as a disease by the International Classification of Disease, Tenth Revision, Clinical Modification (ICD‐10‐CM) since October 2016^15^ and in Australia since July 2019,^16^, ^17^ an increase in knowledge and diagnosis of sarcopenia among health‐care professionals may be expected. However, the current study shows that only a small percentage of health‐care professionals correctly identified sarcopenia as a disease and that the knowledge about the diagnostic strategy is limited. This is in contrast to a study among Dutch health‐care professionals attending a professional development event on sarcopenia, in which 70% stated to know what sarcopenia is and 21% reported to know how to formally diagnose sarcopenia.^9^ However, conclusions that can be drawn from these results are limited as no further questions were asked to assess the actual knowledge.

The current study is the first to report the retention of knowledge after a professional development event on sarcopenia. The fact that knowledge was not retained over 6 months after a single educational event reinforces the need for continuous education to guarantee sufficient knowledge about sarcopenia and to provide evidence‐based practice for optimal patient care. A review about the retention of basic science knowledge in medical school showed that approximately two‐thirds to three‐fourths of knowledge gained via formal education in schools is retained after 1 year.^22^ Systematic reviews examining the effectiveness of professional development events showed that multiple exposures to professional development events were associated with a greater effect on the professionals’ knowledge and performance compared to a single exposure.^23^, ^24^ To promote professional behaviour change in health care and raise the awareness of sarcopenia among health‐care professionals, interventions should combine audits, feedback and reminders in addition to education.^25^ Education should be continuous, implemented more frequently and combined with regular auditing and practice follow‐up in an attempt to sustain knowledge and professional behaviour change.

### 4.2 Sarcopenia diagnosis in clinical practice

Sarcopenia diagnosis in clinical practice was low before attendance. Although an algorithm for sarcopenia diagnosis based on measurements of muscle mass, handgrip strength and gait speed has been suggested by the EWGSOP,^12^ the current study found that these criteria were used the least. Only a few health‐care professionals who diagnosed sarcopenia applied an appropriate sarcopenia definition such as EWGSOP 2010,^12^ IWGS^18^ and Janssen 2004.^19^

In a previous survey among Australian dietitians, 19.0% reported diagnosing sarcopenia and the top three criteria used to diagnose sarcopenia were loss of muscle mass (31%), loss of muscle strength (28%) and weight loss (16%).^20^ The practice of sarcopenia diagnosis differs between countries. Sarcopenia diagnosis was reported by 65.9% of dietitians in Europe.^21^ Among Japanese health‐care professionals (including mainly dietitians and physiotherapists), 41.6% measured all items required for sarcopenia diagnosis, including muscle mass (51.5%), muscle strength (69.1%) and physical function (67.9%).^10^ Although health‐care professionals in the current study had a strong intention to diagnose sarcopenia directly after attendance, the percentage of health‐care professionals who diagnosed sarcopenia remained low 6 months after attendance. This is in contrast to 53.8% of Dutch health‐care professionals who indicated to have implemented the diagnostic strategy in clinical practice.^9^ The difference in the practice of sarcopenia diagnosis may be due to the different health‐care system and awareness among health‐care professionals. Furthermore, clarity between health‐care professionals around responsibilities in diagnosing sarcopenia is warranted. Expectations of who should be diagnosing sarcopenia differ widely among health‐care professionals.

### 4.3 Experienced barriers

The current study found that a lack of diagnostic tools was the main reason for not diagnosing sarcopenia. This is in line with our previous study; the availability of diagnostic tools was the most often‐reported barrier during the implementation of diagnostic strategy among Dutch health‐care professionals.^9^ Lack of awareness and lack of motivation among health‐care professionals were common perceived barriers during sarcopenia treatment in the current study. However, it is known that collaboration between health‐care professionals supports high‐quality and safe care for patients.^26^ Therefore, increasing awareness and motivation among other health‐care professionals within a team is essential. Previously, it was shown that institutes with multidisciplinary teams had a higher proportion of measurements of muscle mass, muscle strength and physical performance for diagnosing sarcopenia.^27^ This highlights the importance of collaboration between health‐care professionals.

Considering the impact of sarcopenia on public health such as high rates of physical disability, nursing home admissions, depression, hospitalisation and mortality, and the associated health‐care costs,^7^ funding and resources from the government and health organisations are required to provide diagnostic tools, manpower and education for effective sarcopenia diagnosis and treatment. For this, a collaboration between health‐care professionals, as well as advocacy from health professional associations, is crucial so that information from those working on the front lines can be delivered to policymakers.^28^, ^29^

### 4.4 Implications

In addition to placing an emphasis on education, a supportive work environment may further enable health‐care professionals to diagnose and treat sarcopenia.^30^ At the individual level, health‐care professionals should actively engage in continuous professional development to acquire up‐to‐date knowledge and collaborate with other health‐care professionals. At the organisation level, funding and resources should be allocated to allow for professional development and the manpower required for sarcopenia diagnosis and treatment.

### 4.5 Strengths and limitations

This is the first study assessing the knowledge and practice of sarcopenia among health‐care professionals after sarcopenia was recognised as a disease in October 2016. In addition, this is the first study to evaluate the retention of knowledge 6 months after a professional development event in sarcopenia. Findings from this study may not be generalisable to the general population of health‐care professionals, as the current population addressed interested professionals who voluntarily signed up for an educational event. Another limitation was the attrition in the response rate 6 months after attending the Sarcopenia Roadshow, and those who responded may have over‐ or underestimated the results. Furthermore, a newly developed custom self‐report questionnaire was used which may have induced socially desirable responses.

## 5 CONCLUSIONS

There is limited knowledge about sarcopenia and its diagnostic strategy among Australian and New Zealand health‐care professionals attending a professional development event on sarcopenia. A single educational event resulted in an improvement in health‐care professionals’ knowledge in this topic, but retention of knowledge remains a challenge to be addressed. Intention to diagnose sarcopenia contrasts the practice in diagnosing sarcopenia. Next to required educational strategies, practical issues have to be resolved to overcome barriers in diagnosing and treating sarcopenia.

## CONFLICT OF INTEREST

The authors declare that they have no conflicts of interest.
