# Supplementary material for: Current knowledge and practice of Australian and New Zealand health‐care professionals in sarcopenia diagnosis and treatment: Time to move forward!
Source: Australas J Ageing. 2019 Oct 15;39(2):e185–93. doi: 10.1111/ajag.12730 (PMC7497106; doi:10.1111/ajag.12730)
Supplement: Supplementary file 1 [file AJAG-39-e185-s001.pdf]

Code: ☐☐☐☐

Date & Location: ☐ 25/09/2017 - Auckland, NZ ☐ 26/09/2017 - Tauranga, NZ
☐ 27/09/2017 - Palmerston, NZ ☐ 28/09/2017- Christchurch, NZ
☐ 09/10/2017 - Melbourne, AUS ☐ 10/10/2017 - Sydney, AUS

Before attendance

Before attending the Sarcopenia Roadshow, we invite you to complete this questionnaire about your background, current practice and knowledge about sarcopenia. Your responses will provide valuable information for us to design future sarcopenia-related education.
The questionnaire will take no longer than 3 minutes.

General
**1.1 What is your age?** ☐☐ years
**1.2 What is your sex?** ☐ Male ☐ Female
**1.3 What is your current profession?**<br>☐ Dietitian → Grade: ☐ 1 ☐ 2 ☐ 3 ☐ 4 ☐ Not applicable<br>☐ Exercise physiologist<br>☐ Medical doctor → ☐ General practitioner<br>☐ Intern/Resident<br>☐ Registrar →Please indicate your speciality:<br>☐ Consultant →Please indicate your speciality:<br>☐ Cardiology ☐ Endocrinology<br>☐ General medicine ☐ Geriatrics<br>☐ Neurology ☐ Oncology<br>☐ Others, please specify: ____________<br>☐ Nurse → ☐ Registered nurse ☐ Enrolled nurse<br>☐ Nurse practitioner<br>☐ Occupational therapist → Grade: ☐ 1 ☐ 2 ☐ 3 ☐ 4 ☐ Not applicable<br>☐ Physiotherapist → Grade: ☐ 1 ☐ 2 ☐ 3 ☐ 4 ☐ Not applicable<br>☐ Podiatrist → Grade: ☐ 1 ☐ 2 ☐ 3 ☐ 4 ☐ Not applicable<br>☐ Others, please specify: ______________________________
**1.4 How many years have you worked in your current profession?**<br>☐☐ years ☐☐ months
**1.5 Which setting do you work in?**<br>☐ Community service ☐ General practice ☐ Outpatient clinic ☐ Nursing home<br>☐ Hospital → ☐ acute ☐ subacute<br>☐ Others, please specify: ______________________
**1.6 Do you work with patients aged 60 years or older?** ☐ Yes ☐ No
**1.7 Have you received any sarcopenia-related education in the last 6 months?**<br>☐ Yes → multiple answers possible ☐ Seminar/Workshop ☐ Conference<br>☐ Online training<br>☐ Others, please specify: __________________<br>☐ No

Knowledge about sarcopenia
Below are questions relating to sarcopenia, please mark the best option based on your current knowledge:
**1.8 Sarcopenia is recognised as a ...**<br>☐ Disease ☐ Syndrome ☐ Condition ☐ Don't know
**1.9 Sarcopenia cannot be prevented.**<br>☐ Agree ☐ Disagree ☐ Don't know
**1.10 Overweight or obese individuals have lower risk of sarcopenia compared to individuals with normal body weight.**<br>☐ Agree ☐ Disagree ☐ Don't know
**1.11 Which criteria should be used to diagnose sarcopenia (multiple answers possible)?**<br>☐ Clinical impression<br>☐ Muscle mass<br>☐ Muscle strength<br>☐ Physical performance, e.g. walking speed<br>☐ Nutritional status<br>☐ Body mass index<br>☐ Frailty criteria<br>☐ Others, please specify: ______________________________
**1.12 Muscle mass and strength peak in early adulthood, followed by a gradual decline at the age of ☐☐ years old.**
**1.13 According to the European Working Group on Sarcopenia in Older Persons (EWGSOP), low handgrip strength is defined as less than:**<br>**☐☐ kg for males;**<br>**☐☐ kg for females.**
**1.14 Sarcopenia should be treated with (multiple answers possible)…**<br>☐ Physical Exercise → ☐ Aerobic ☐ Resistance ☐ Balance<br>☐ Nutritional intervention → ☐ Protein ☐ Vitamin D ☐ Calcium<br>☐ Pharmacological intervention<br>☐ Don't know

Current practice
**1.15 Have you diagnosed sarcopenia in your practice in the previous month?**<br>☐ Yes<br>☐ No → Reason(s): ☐ I do not work with older adults aged 60 years or older.<br>☐ I do not have the tools to diagnose sarcopenia.<br>☐ I am not responsible for diagnosing sarcopenia.<br>☐ Others, please specify:________________________________<br>________________________________<br>→ skip to question 1.20
**1.16 How did you diagnose sarcopenia (multiple answers possible)?**<br>☐ Clinical impression<br>☐ Muscle mass → tools used:<br>☐ Calf circumference ☐ Skinfold thickness ☐ DXA<br>☐ Bioelectrical impedance analyser (BIA) → ☐ Single frequency ☐ Multi-frequency<br>☐ Don't know<br>☐ Imaging → ☐ MRI ☐ CT<br>☐ Others, please specify: ____________________<br>☐ Muscle strength → tools used:<br>☐ Handgrip dynamometer ☐ Isokinetic dynamometer ☐ Leg press<br>☐ Others, please specify: ____________________<br>☐ Physical performance → tools used:<br>☐ Gait speed ☐ Sit to stand/ Chair stand ☐ Timed Up & Go<br>☐ Short Physical Performance Battery (SPPB)<br>☐ Others, please specify: ____________________<br>☐ Nutritional status → tools used:<br>☐ Malnutrition Screening Tool (MST) ☐ Mini-Nutritional Assessment (MNA)<br>☐ Subjective Global Assessment<br>☐ Others, please specify: ____________________<br>☐ Body mass index<br>☐ Frailty criteria<br>☐ Others, please specify: ________________________________
**1.17 Which definition have you applied to diagnose sarcopenia?**<br>☐ European Working Group on Sarcopenia in Older Persons (EWGSOP)<br>☐ International Working Group on Sarcopenia (IWGS)<br>☐ Foundation for the National Institutes of Health (FNIH)<br>☐ Appendicular lean mass Index by Baumgartner 1998<br>☐ Skeletal Muscle Mass Index by Janssen 2004<br>☐ European Society for Clinical Nutrition and Metabolism (ESPEN) definition of malnutrition<br>☐ Frailty criteria by Fried<br>☐ Frailty criteria by Rockwood<br>☐ Others, please specify: ________________________________

1.18 In which individuals do you apply the diagnostic measures to diagnose sarcopenia (multiple answers possible)?

- ☐ All older adults
- ☐ Older adults with comorbidity
- ☐ Older adults with mobility problems
- ☐ Older adults with malnutrition
- ☐ Others, please specify: ______________________________

1.19 Have you documented the diagnosis of sarcopenia in the medical record?

☐ Always ☐ Sometimes ☐ Never

1.20 Are you responsible for providing treatment for patients diagnosed with sarcopenia (multiple answers possible)?

- ☐ Yes → ☐ Physical exercise → ☐ Aerobic ☐ Resistance ☐ Balance
  - ☐ Nutrition → ☐ Protein ☐ Vitamin D ☐ Calcium
  - ☐ Others, please specify: ______________________________
- ☐ No

1.21 Do you consult other disciplines when you have a patient diagnosed with sarcopenia (multiple answers possible)?

- ☐ Yes
- ☐ No → skip to Question 1.23

1.22 Which discipline(s) do you consult when you have a patient diagnosed with sarcopenia (multiple answers possible)?

- ☐ Dietitian
- ☐ Exercise physiologist
- ☐ Medical doctor → ☐ General Practitioner ☐ Specialist, please specify: ____________
- ☐ Nurse
- ☐ Nurse practitioner
- ☐ Occupational therapist
- ☐ Physiotherapist
- ☐ Podiatrist
- ☐ Others, please specify: ____________________

1.23 Is there a protocol for <u>diagnosing</u> sarcopenia in your workplace?

☐ Yes ☐ No ☐ Don't know

1.24 Is there a protocol for <u>treating</u> sarcopenia in your workplace?

☐ Yes ☐ No ☐ Don't know

Code: ☐☐☐☐

Date & Location: ☐ 25/09/2017 - Auckland, NZ ☐ 26/09/2017 - Tauranga, NZ
☐ 27/09/2017 - Palmerston, NZ ☐ 28/09/2017- Christchurch, NZ
☐ 09/10/2017 - Melbourne, AUS ☐ 10/10/2017 - Sydney, AUS

Directly after attendance

Thank you for attending the Sarcopenia Roadshow. Please take 3 minutes to complete this questionnaire about your intention for change in practice and knowledge about sarcopenia.

**Knowledge about sarcopenia**
Below are questions relating to sarcopenia, please mark the best option based on your current knowledge:
**2.1 Sarcopenia is recognised as a ...**<br>☐ Disease ☐ Syndrome ☐ Condition ☐ Don't know
**2.2 Sarcopenia cannot be prevented.**<br>☐ Agree ☐ Disagree ☐ Don't know
**2.3 Overweight or obese individuals have lower risk of sarcopenia compared to patients with normal body weight.**<br>☐ Agree ☐ Disagree ☐ Don't know
**2.4 Which criteria should be used to diagnose sarcopenia (multiple answers possible)?**<br>☐ Clinical impression<br>☐ Muscle mass<br>☐ Muscle strength<br>☐ Physical performance, e.g. walking speed<br>☐ Nutritional status<br>☐ Body mass index<br>☐ Frailty criteria<br>☐ Others, please specify: ________________
**2.5 Muscle mass and strength peak in early adulthood, followed by a gradual decline at the age of ☐☐ years old.**
**2.6 According to the European Working Group on Sarcopenia in Older Persons (EWGSOP), low handgrip strength is defined as less than:**<br>**☐☐ kg for males;**<br>**☐☐ kg for females.**
**2.7 Sarcopenia should be treated with (multiple answers possible)…**<br>☐ Physical exercise → ☐ Aerobic ☐ Resistance ☐ Balance<br>☐ Nutritional intervention → ☐ Protein ☐ Vitamin D ☐ Calcium<br>☐ Pharmacological intervention<br>☐ Don't know

Intention to change practice

2.8 Do you intend to diagnose sarcopenia in your practice?

☐ Not applicable, I already diagnose sarcopenia in my current practice.
☐ Yes
☐ No → Reason(s): ☐ I do not work with older adults aged 60 years or older.
☐ I do not have the tools to diagnose sarcopenia.
☐ I am not responsible for diagnosing sarcopenia.
☐ Others, please specify:______________________________
____

→ skip to question 2.13

2.9 Which diagnostic measure(s) do you intend to use to diagnose sarcopenia (multiple answers possible)?

☐ Clinical impression
☐ Muscle mass → tools used:
☐ Calf circumference ☐ Skinfold thickness ☐ DXA
☐ Bioelectrical impedance analyser (BIA) → ☐ Single frequency ☐ Multi-frequency ☐ Don't know
☐ Imaging → ☐ MRI ☐ CT
☐ Others, please specify: ___________________
☐ Muscle strength → tools used:
☐ Handgrip dynamometer ☐ Isokinetic dynamometer ☐ Leg press
☐ Others, please specify: ___________________
☐ Physical performance → tools used:
☐ Gait speed ☐ Sit to stand/Chair stand ☐ Timed Up & Go
☐ Short Physical Performance Battery (SPPB)
☐ Others, please specify: ___________________
☐ Nutritional status → tools used:
☐ Malnutrition Screening Tool (MST) ☐ Mini-Nutritional Assessment (MNA)
☐ Subjective Global Assessment
☐ Others, please specify: ___________________
☐ Body mass index
☐ Frailty criteria
☐ Others, please specify: ____

2.10 Do you intend to document the diagnosis of sarcopenia in the medical record?

☐ Yes ☐ No ☐ Don't know

2.11 Do you intend to consult other discipline(s) when you diagnose sarcopenia?

☐ Yes
☐ No → skip to question 2.13

2.12 Which discipline(s) do you intend to <u>consult</u> when you diagnose sarcopenia (multiple answers allowed)?

- ☐ Dietitian
- ☐ Exercise physiologist
- ☐ Medical doctor → ☐ General practitioner ☐ Specialist, please specify:____________
- ☐ Nurse
- ☐ Nurse practitioner
- ☐ Occupational therapist
- ☐ Physiotherapist
- ☐ Podiatrist
- ☐ Others, please specify: __________________

2.13 Do you intend to identify/assist in developing a protocol for <u>diagnosing</u> sarcopenia in your workplace?

- ☐ Yes
- ☐ No
- ☐ Not applicable, there is already a protocol in my workplace.

2.14 Do you intend to identify/assist in developing a protocol for <u>treating</u> sarcopenia in your workplace?

- ☐ Yes
- ☐ No
- ☐ Not applicable, there is already a protocol in my workplace.

2.15 Do you intend to <u>collaborate with</u> other discipline(s) to provide treatment for patients diagnosed with sarcopenia?

- ☐ Yes
- ☐ No → skip to question 2.17

2.16 Which discipline(s) do you intend to <u>collaborate with</u> to provide treatment for patients diagnosed with sarcopenia (multiple answers possible)?

- ☐ Dietitian
- ☐ Exercise physiologist
- ☐ Medical doctor → ☐ General practitioner ☐ Specialist, please specify:____________
- ☐ Nurse
- ☐ Nurse practitioner
- ☐ Occupational therapist
- ☐ Physiotherapist
- ☐ Podiatrist
- ☐ Others, please specify: ____________________

2.17 Do you intend to recommend other discipline(s) to receive sarcopenia-related education?

- ☐ Yes
- ☐ No → skip to 2.19

2.18 Which discipline(s) do you intend to recommend to receive sarcopenia-related education (multiple answers possible)?

☐ Dietitian
☐ Exercise physiologist
☐ Medical doctor → ☐ General practitioner ☐ Specialist, please specify: ___________
☐ Nurse
☐ Nurse practitioner
☐ Occupational therapist
☐ Physiotherapist
☐ Podiatrist
☐ Others, please specify: ___________________

2.19 We would like to contact you in five months by email to complete a follow-up online questionnaire. Your responses will help us to understand possible barriers you are facing when diagnosing and treating sarcopenia.

☐ Yes, please contact me.
First and last name: _

Email address: _

☐ No

Thanks in advance for your help!

Six months after attendance

**You attended one of our Sarcopenia Roadshow six months ago. We would be grateful if you would complete this follow-up questionnaire regarding your current practice and barriers encountered whilst diagnosing and treating sarcopenia.**

**Knowledge about sarcopenia**
The following are questions relating to sarcopenia. Based on your current knowledge, please indicate the most accurate answer.
**3.1 Sarcopenia is recognised as a ...**<br>☐ Disease ☐ Syndrome ☐ Condition ☐ Unsure
**3.2 Sarcopenia cannot be prevented.**<br>☐ Agree ☐ Disagree ☐ Unsure
**3.3 Overweight or obese individuals have a lower risk of sarcopenia compared to patients with normal body weight.**<br>☐ Agree ☐ Disagree ☐ Unsure
**3.4 Which criteria should be used to diagnose sarcopenia (multiple answers possible)?**<br>☐ Clinical impression<br>☐ Muscle mass<br>☐ Muscle strength<br>☐ Physical performance, e.g. walking speed<br>☐ Nutritional status<br>☐ Body mass index<br>☐ Frailty criteria<br>☐ Other, please specify: ______________________________
**3.5 At what age does muscle mass and muscle strength start to decline?**<br>☐☐ **years old.**
**3.6 What is the cut-off for low handgrip strength?**<br>less than ☐☐ kg for males;<br>less than ☐☐ kg for females.<br>☐ Unsure
**3.7 Sarcopenia should be treated with (multiple answers possible)…**<br>☐ Physical exercise → ☐ Aerobic ☐ Resistance ☐ Balance<br>☐ Nutritional intervention → ☐ Protein ☐ Vitamin D ☐ Calcium<br>☐ Pharmacological intervention<br>☐ Unsure

Current practice

3.8 Have you diagnosed sarcopenia in your practice since the Sarcopenia Roadshow?

☐ Yes

☐ No → Reason(s):
- ☐ I do not work with older adults aged 60 years or older.
- ☐ I do not have the tools to diagnose sarcopenia.
- ☐ I am not responsible for diagnosing sarcopenia.
- ☐ Other, please specify:______________________________

____

No → skip to question 3.16

3.9 How have you diagnosed sarcopenia (multiple answers possible)?

☐ Clinical impression

☐ Muscle mass → tools used:
- ☐ Calf circumference ☐ Skinfold thickness ☐ DXA
- ☐ Bioelectrical impedance analyser (BIA)→ ☐ Single frequency ☐ Multi-frequency ☐ Unsure
- ☐ Imaging → ☐ MRI ☐ CT
- ☐ Other, please specify: ___________________

☐ Muscle strength → tools used:
- ☐ Handgrip dynamometer ☐ Isokinetic dynamometer ☐ Leg press
- ☐ Other, please specify: ___________________

☐ Physical performance → tools used:
- ☐ Gait speed ☐ Sit to stand/Chair stand ☐ Timed Up & Go
- ☐ Short Physical Performance Battery (SPPB)
- ☐ Other, please specify: ___________________

☐ Nutritional status → tools used:
- ☐ Malnutrition Screening Tool (MST) ☐ Mini-Nutritional Assessment (MNA)
- ☐ Subjective Global Assessment
- ☐ Other, please specify: ___________________

☐ Body mass index

☐ Frailty criteria

☐ Other, please specify: ____

3.10 Which definition(s) have you applied to diagnose sarcopenia?

☐ European Working Group on Sarcopenia in Older Persons (EWGSOP)

☐ International Working Group on Sarcopenia (IWGS)

☐ Foundation for the National Institutes of Health (FNIH)

☐ Appendicular lean mass Index by Baumgartner 1998

☐ Skeletal Muscle Mass Index by Janssen 2004

☐ European Society for Clinical Nutrition and Metabolism (ESPEN) definition of malnutrition

☐ Frailty criteria by Fried

☐ Frailty criteria by Rockwood

☐ Other, please specify: _______________________________________

3.11 In which group(s) have you applied the above diagnostic measures to confirm sarcopenia (multiple answers possible)?

- ☐ All older adults
- ☐ Older adults with comorbidity
- ☐ Older adults with mobility problems
- ☐ Older adults with malnutrition
- ☐ Other, please specify: ____

3.12 Which discipline(s) have you <u>collaborated with</u> when diagnosing sarcopenia (multiple answers possible)?

- ☐ Dietitian
- ☐ Exercise physiologist
- ☐ Medical doctor → ☐ General practitioner ☐ Specialist, please specify: ________
- ☐ Nurse
- ☐ Nurse practitioner
- ☐ Occupational therapist
- ☐ Physiotherapist
- ☐ Podiatrist
- ☐ Other, please specify: __________________
- ☐ None

3.13 Have you experienced any barrier(s) when diagnosing sarcopenia?

- ☐ Yes
- ☐ No → skip to question 3.15

3.14 What barrier(s) have you experienced when diagnosing sarcopenia (multiple answers possible)?

- ☐ Lack of awareness among other healthcare professionals
- ☐ Acquisition of a device to measure muscle mass
- ☐ I am not trained to measure muscle mass
- ☐ Acquisition of handgrip strength device
- ☐ I do not have the skill in measuring handgrip strength
- ☐ No space for walking test
- ☐ Time constraints to perform the diagnostic tests
- ☐ No funding source specific for sarcopenia
- ☐ Other, please specify: ____

3.15 Have you documented the diagnosis of sarcopenia in the medical record?

☐ Always ☐ Sometimes ☐ Never

3.16 When you diagnosed a patient with sarcopenia, did you consult other discipline?

- ☐ No
- ☐ Yes →
  - ☐ Dietitian
  - ☐ Exercise physiologist
  - ☐ Medical doctor → ☐ General practitioner ☐ Specialist, please specify: ________
  - ☐ Nurse
  - ☐ Nurse practitioner
  - ☐ Occupational therapist
  - ☐ Physiotherapist
  - ☐ Podiatrist
  - ☐ Other, please specify: __________________

3.17 Since you attended the Sarcopenia Roadshow, have you identified or assisted in developing a protocol for <u>diagnosing</u> sarcopenia in your workplace?

- ☐ Yes
- ☐ No
- ☐ Not applicable, there is already a protocol in my workplace.

3.18 Since you attended the Sarcopenia Roadshow, have you identified or assisted in developing a protocol for <u>treating</u> sarcopenia in your workplace?

- ☐ Yes
- ☐ No
- ☐ Not applicable, there is already a protocol in my workplace.

3.19 Are you responsible for providing treatment to patients diagnosed with sarcopenia (multiple answers possible)?

- ☐ Yes → ☐ Physical exercise → ☐ Aerobic ☐ Resistance ☐ Balance
  - ☐ Nutrition → ☐ Protein ☐ Vitamin D ☐ Calcium
  - ☐ Other, please specify: ____
- ☐ No

3.20 Did you <u>collaborate with</u> other discipline(s) to provide <u>treatment</u> to patients diagnosed with sarcopenia (multiple answers possible)?

- ☐ No
- ☐ Yes →
  - ☐ Dietitian
  - ☐ Exercise physiologist
  - ☐ Medical doctor → ☐ General practitioner ☐ Specialist, please specify: ________
  - ☐ Nurse
  - ☐ Nurse practitioner
  - ☐ Occupational therapist
  - ☐ Physiotherapist
  - ☐ Podiatrist
  - ☐ Other, please specify: ____________________

Questions 3.21-3.22 are related to the <u>implementation phase of your sarcopenia treatment plan</u>:

3.21 Have you experienced any barrier(s) during the implementation of your sarcopenia treatment plan?

- ☐ Yes
- ☐ No → skip to question 3.23

3.22 What barrier(s) have you experienced during the implementation of your sarcopenia treatment plan (multiple answers possible)?

- ☐ Re-structuring of routine care
- ☐ Lack of awareness among other healthcare professionals
- ☐ Lack of collaboration with other healthcare professionals
- ☐ There is no protocol for treating sarcopenia in my workplace
- ☐ Sarcopenia is not a priority for my discipline/other healthcare professionals
- ☐ Patients refused to be treated
- ☐ Patients are not aware of the importance to treat sarcopenia
- ☐ Other, please specify: ____

Questions 3.23-3.24 are related to the actual treatment provided to your patients.
3.23 Have you experienced any barrier(s) in treating patients with sarcopenia?

- ☐ Yes
- ☐ No → skip to question 3.25

3.24 What barrier(s) have you experienced in treating patients with sarcopenia (multiple answers possible)?

- ☐ I do not have access to other professional with experience treating sarcopenia
- ☐ Lack of awareness among other healthcare professionals
- ☐ Lack of motivation in treating sarcopenia among other healthcare professionals
- ☐ I am not motivated to treat patients with sarcopenia
- ☐ Patients are not motivated to be treated
- ☐ Patients not compliant with treatment plan
- ☐ Financial implications to patient for treatment
- ☐ Not enough manpower to treat sarcopenia
- ☐ Other, please specify: ______________________________

3.25 Did you receive our sarcopenia pocket card?

- ☐ Yes
- ☐ No → skip to 3.28

3.26 Did you use the sarcopenia pocket card in your clinical practice?

- ☐ Yes
- ☐ No, reason: ______________________________

3.27 What information would you suggest be added to/removed from the sarcopenia pocket card?

3.28 What else do you need to more effectively diagnose sarcopenia in your clinical practice?

3.29 What else do you need to more effectively treat sarcopenia in your clinical practice?
