# Supplementary material for: Current knowledge and practice of Australian and New Zealand health‐care professionals in sarcopenia diagnosis and treatment: Time to move forward!
Source: Australas J Ageing. 2019 Oct 15;39(2):e185–93. doi: 10.1111/ajag.12730 (PMC7497106; doi:10.1111/ajag.12730)
Supplement: Supplementary file 2 [file AJAG-39-e185-s002.pdf]

Appendix II. Characteristics of healthcare professionals stratified by the level of knowledge† about sarcopenia before attendance of the Sarcopenia Roadshow

	**Better knowledge (n=64)**	**Worse knowledge (n=177)**	**P**
Age, years, median [IQR]	48 [31-55]	38 [27-55]	0.224
Female	53 (86.9)	147 (84.0)	0.589
Profession			0.224
Dietitian	43 (67.2)	101 (57.1)	
Medical doctor	8 (12.5)	44 (24.9)	
Nurse/nurse practitioner	10 (15.6)	26 (14.7)	
Others	3 (4.7)	6 (3.4)	
Year of practice, median [IQR]	16 [4-30]	10 [3-30]	0.253
Setting			
Community service	18 (29.0)	17 (9.7)	**<0.001**
General practice	11 (17.7)	50 (28.6)	0.094
Outpatient clinic	8 (12.9)	15 (8.6)	0.322
Nursing home	7 (11.3)	13 (7.4)	0.347
Hospital	33 (53.2)	97 (55.4)	0.765
Other settings	7 (11.3)	22 (12.6)	0.791
Work with patients aged ≥65 years, yes	61 (97.8)	169 (97.7)	0.710
Received sarcopenia-related education in the past 6 months	16 (25.4)	24 (13.8)	**0.035**

Variables are presented as n (%) unless indicated otherwise

†Healthcare professionals were divided into quartiles and characteristics of the 25% with the best knowledge were compared to the 75% with worse knowledge about sarcopenia.
