# Supplementary figures and images for: Current knowledge and practice of Australian and New Zealand health‐care professionals in sarcopenia diagnosis and treatment: Time to move forward!
Source: Australas J Ageing. 2019 Oct 15;39(2):e185–93. doi: 10.1111/ajag.12730 (PMC7497106; doi:10.1111/ajag.12730)

### Appendix III

#### Before attendance

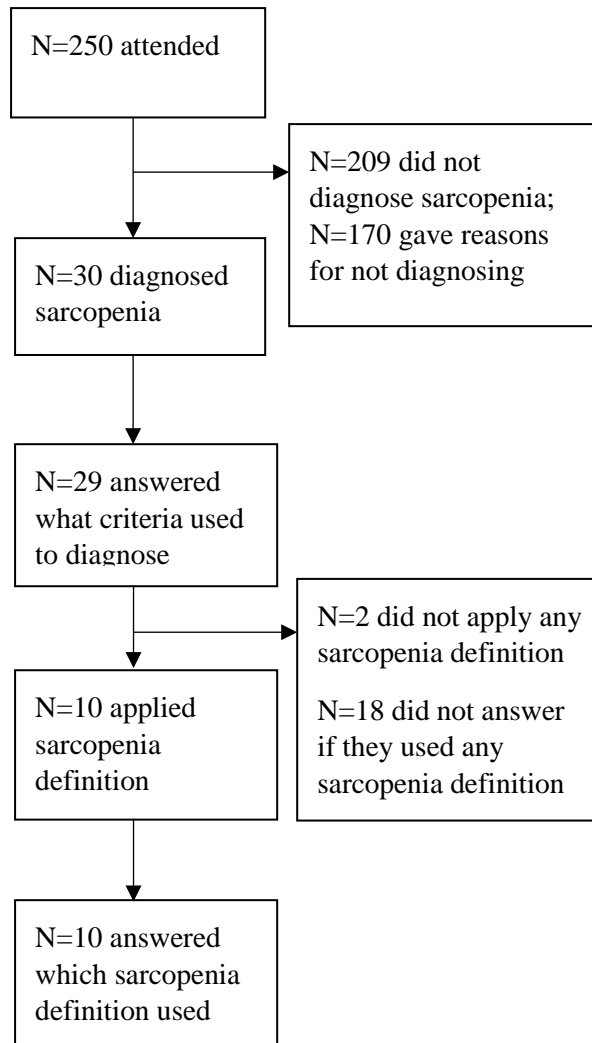

#### Directly after attendance

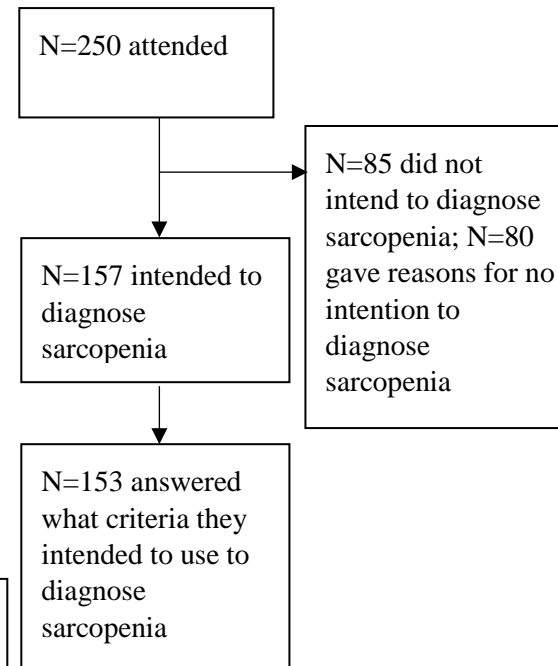

#### Six months after attendance

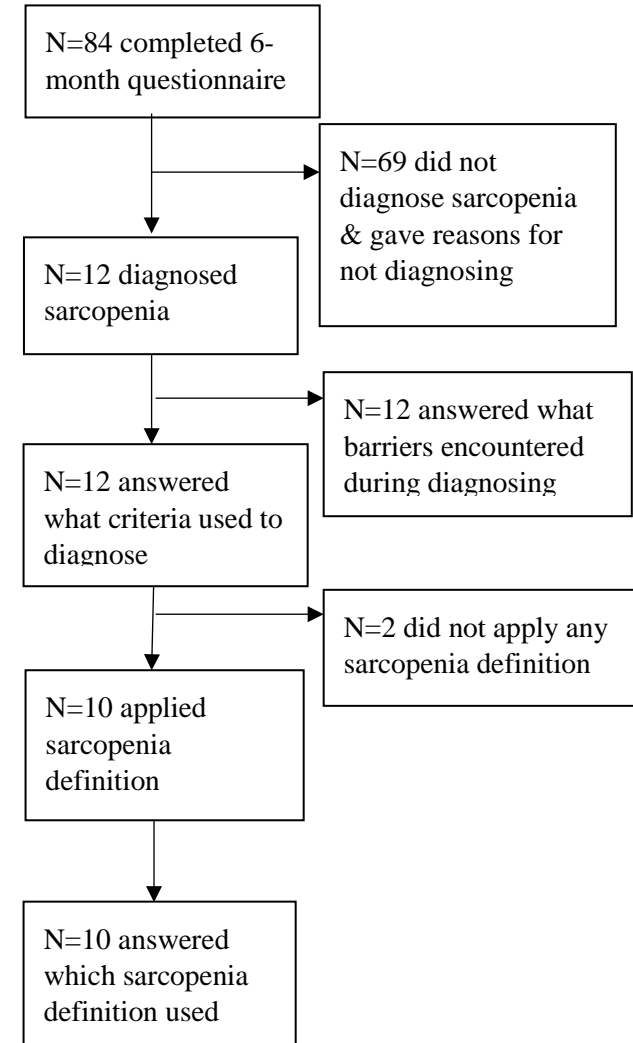

Supplement: Supplementary file 3 [file AJAG-39-e185-s003.pdf]
