# Supplementary material for: Current knowledge and practice of Australian and New Zealand health‐care professionals in sarcopenia diagnosis and treatment: Time to move forward!
Source: Australas J Ageing. 2019 Oct 15;39(2):e185–93. doi: 10.1111/ajag.12730 (PMC7497106; doi:10.1111/ajag.12730)

Appendix IV

Before attendance:

a. Diagnostic criteria used
(multiple answers possible)

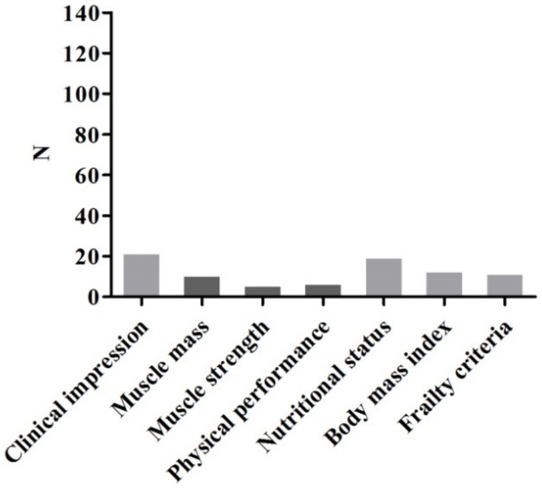

b. Definition used
(multiple answers possible)

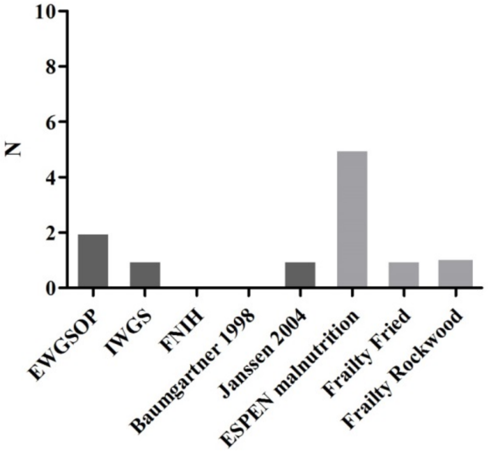

Directly after attendance:

a. Diagnostic criteria intended to use
(multiple answers possible)

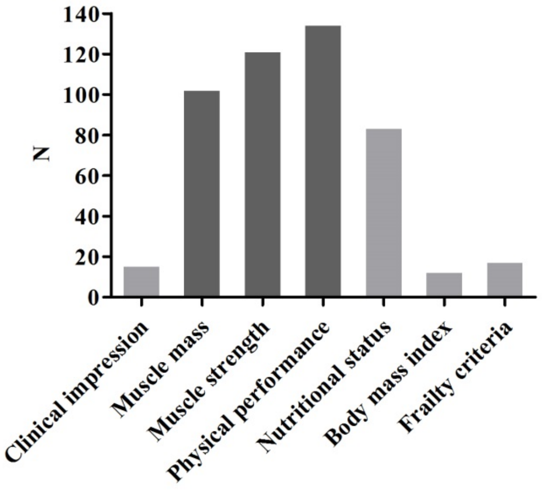

Six months after attendance:

a. Diagnostic criteria used
(multiple answers possible)

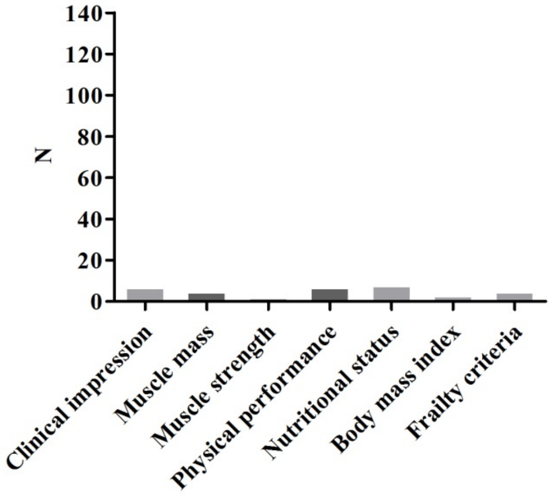

b. Definition used
(multiple answers possible)

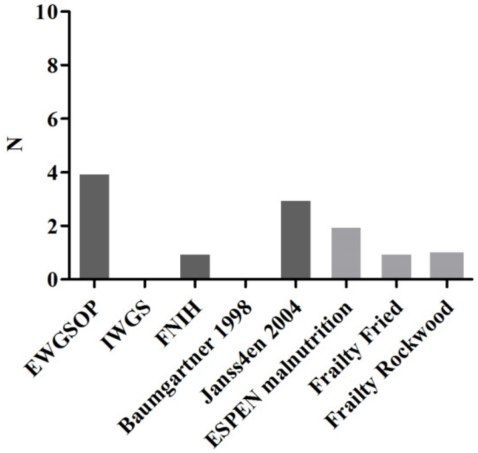

Supplement: Supplementary file 4 [file AJAG-39-e185-s004.pdf]
